# Hydrogen Sulfide Attenuates Angiotensin II-Induced Cardiac Fibroblast Proliferation and Transverse Aortic Constriction-Induced Myocardial Fibrosis through Oxidative Stress Inhibition via Sirtuin 3

**DOI:** 10.1155/2021/9925771

**Published:** 2021-09-23

**Authors:** Lulu Liu, Weiwei Gong, Shuping Zhang, Jieru Shen, Yuqin Wang, Yun Chen, Guoliang Meng

**Affiliations:** ^1^Department of Pharmacology, School of Pharmacy, Nantong University, Nantong, 226001 Jiangsu, China; ^2^Department of Pharmacy, Affiliated Maternity & Child Health Care Hospital of Nantong University, Nantong 226001, China

## Abstract

Sirtuin 3 (SIRT3) is critical in mitochondrial function and oxidative stress. Our present study investigates whether hydrogen sulfide (H_2_S) attenuated myocardial fibrosis and explores the possible role of SIRT3 on the protective effects. Neonatal rat cardiac fibroblasts were pretreated with NaHS followed by angiotensin II (Ang II) stimulation. SIRT3 was knocked down with siRNA technology. SIRT3 promoter activity and expression, as well as mitochondrial function, were measured. Male wild-type (WT) and SIRT3 knockout (KO) mice were intraperitoneally injected with NaHS followed by transverse aortic constriction (TAC). Myocardium sections were stained with Sirius red. Hydroxyproline content, collagen I and collagen III, *α*-smooth muscle actin (*α*-SMA), and dynamin-related protein 1 (DRP1) expression were measured both *in vitro* and *in vivo*. We found that NaHS enhanced SIRT3 promoter activity and increased SIRT3 mRNA expression. NaHS inhibited cell proliferation and hydroxyproline secretion, decreased collagen I, collagen III, *α*-SMA, and DRP1 expression, alleviated oxidative stress, and improved mitochondrial respiration function and membrane potential in Ang II-stimulated cardiac fibroblasts, which were unavailable after SIRT3 was silenced. *In vivo*, NaHS reduced hydroxyproline content, ameliorated perivascular and interstitial collagen deposition, and inhibited collagen I, collagen III, and DRP1 expression in the myocardium of WT mice but not SIRT3 KO mice with TAC. Altogether, NaHS attenuated myocardial fibrosis through oxidative stress inhibition via a SIRT3-dependent manner.

## 1. Introduction

Myocardial fibrosis is a cardiac interstitial remodeling characterized by excessive cardiac fibroblast proliferation, collagen deposition, and abnormal distribution [1, 2]. It is closely related to hypertension, chronic heart failure, hypertrophic cardiomyopathy, dilated cardiomyopathy, viral myocarditis, and many other cardiovascular diseases, which is a potential risk factor of sudden cardiac death [3–7]. However, there is no ideal strategy for the treatment of myocardial fibrosis.

Hydrogen sulfide (H_2_S) is considered to be the third gasotransmitter after nitric oxide (NO) and carbon monoxide (CO) [8, 9]. Endogenous H_2_S is catalyzed by cystathionine-*β*-synthase (CBS), cystathionine-*γ*-lyase (CSE), 3-mercaptopyruvate sulfurtransferase (3-MST), and so on [10, 11]. The enzymes are tissue-specific distributed in different systems. CBS is a key enzyme to produce H_2_S in the nervous system, while CSE is mainly in the cardiovascular system [12, 13]. Previous studies verified that H_2_S had protective effects against atherosclerosis, myocardial hypertrophy, myocardial ischemia-reperfusion injury, endothelial cell damage, spermatogenic failure, and testicular dysfunction [13–21]. Moreover, we also found that H_2_S donor GYY4137 alleviated myocardial fibrosis in spontaneously hypertensive rats [22]. However, the detailed mechanism by which H_2_S attenuated cardiac fibroblast proliferation *in vitro* and myocardial fibrosis *in vivo* remains unclear.

Silent information regulator 2 (SIR2), a highly conserved NAD-dependent family histone deacetylase, serves as a cell sensor for energy utilization and metabolism regulation [23, 24]. SIR2 is widely found in mammals and consists of seven members including sirtuin 1 (SIRT1) to sirtuin 7 (SIRT7). The SIR2 family plays a role in metabolism, cancer, and other physiological and pathological processes [25, 26]. Sirtuin 3 (SIRT3) is a member of histone deacetylase III to mediate redox signaling [27]. Previous research has demonstrated that H_2_S was capable of increasing SIRT3 to improve mitochondrial function and attenuate oxidative stress. We found that H_2_S improved endothelium-dependent relaxation of aortic and mesenteric arteries in paraquat-administrated wild-type (WT) mice but not SIRT3 knockout (KO) mice [18]. H_2_S reduced superoxide anion production in angiotensin II- (Ang II-) stimulated cardiomyocytes, but this effect is significantly weakened after SIRT3 was knocked down. H_2_S protected against myocardial hypertrophy in WT mice but not SIRT3 KO mice [16]. However, it is not known whether H_2_S protects against cardiac fibroblast proliferation and myocardial fibrosis via SIRT3 activation.

Therefore, our present study investigates whether H_2_S attenuated cardiac fibroblast proliferation *in vitro* and myocardial fibrosis *in vivo* and explores the possible role of SIRT3 on the protective effects.

## 2. Materials and Methods

### 2.1. Cardiac Fibroblast Culture and Treatment

After anesthesia by isoflurane, neonatal Sprague Dawley rats aged 1 to 3 days old were killed by decapitation. Then, the left ventricular myocardium was cut into small pieces followed by digestion with trypsin in a constant temperature water bath of 37°C for 5-8 minutes. After about 10 times, all supernatants except the first time were mixed. Next, Dulbecco's modified Eagle's medium (DMEM, Wisent Inc., Montreal, QC, Canada) containing 10% fetal bovine serum (FBS, Wisent Inc., Montreal, QC, Canada) was timely added to stop excessive digestion. After centrifugation, fresh DMEM containing 10% FBS was used to resuspend the cell precipitate. After differential adhesion for 4 h, the cardiomyocytes in the medium were abandoned while confluent cardiac fibroblasts adhered to the walls. Cardiac fibroblasts of the 2^nd^ or 3^rd^ passage were used in our further experiments. Cells were then administrated with NaHS (50 *μ*M, Sigma-Aldrich, St. Louis, MO), a common H_2_S donor, for 4 h followed by Ang II (100 nM, Sigma-Aldrich, St. Louis, MO) stimulation for an additional 24 h.

### 2.2. Cell Proliferation and Hydroxyproline Content Measurement

Cell proliferation was represented as cardiac fibroblast number enhancement, which was determined by Cell Counting Kit-8 (CCK-8, Beyotime, Shanghai, China) as the previous study [4]. The content of hydroxyproline in the cell culture medium and in the myocardium was detected according to our previous research [4, 22].

### 2.3. Luciferase Reporter Assay

Neonatal rat cardiac fibroblasts were transfected with the SIRT3 promoter luciferase fusion plasmid using the Lipofectamine 3000 reagent (Invitrogen, Carlsbad, CA, USA), and the pRL-TK reporter plasmid served as a control reporter. After changing by the fresh medium with 10% FBS 24 h later, cells were incubated with NaHS (50 *μ*M) for 4 h and subsequently stimulated by Ang II (100 nM) for 24 h. Next, the cell lysis buffer was added to harvest the cells. The luciferase activity was detected with a dual-luciferase reporter assay system (Promega, Madison, WI, USA). Activity of the SIRT3 promoter was assessed by the relative luciferase activities compared with pRL-TK, which was normalized to control in triplicate.

### 2.4. SIRT3 RNA Interference

After starvation for 24 h, the mixture of the SIRT3 siRNA or nonspecific control siRNA (NC siRNA) and the Lipofectamine 3000 reagent was added into cardiac fibroblasts. SIRT3 siRNA (5′-CCAUCUUUGAACUAGGCUUTT-3′ and 5′-AAGCCUAGUUCAAAGAUGGTT-3′) or NC siRNA with random sequences was commercially synthesized (GenePharma, Shanghai, China). The expression of SIRT3 mRNA and protein was measured by real-time PCR and western blot, respectively, to evaluate the efficiency of SIRT3 siRNA transfection after 48 h. Some other cells were stimulated with NaHS (50 *μ*M) for 4 h followed by Ang II (100 nM) stimulation for 24 h after siRNA was transfected into the cardiac fibroblasts.

### 2.5. Immunofluorescence

After treatment, cardiac fibroblasts were fixed by polyformaldehyde for 10 min followed by incubation with phosphate-buffered solution (PBS) containing 0.3% Triton X-100 at room temperature for 20 min. The anti-*α*-smooth muscle actin (*α*-SMA, 1 : 50; Bioss, Beijing, China) or anti-dynamin-related protein 1 (DRP1, 1 : 50; Cell Signaling Technology, Danvers, MA, USA) antibody was added into cells at 4°C overnight followed by Cy3- or Alexa Fluor 488-conjugated IgG (1 : 500; Beyotime, Shanghai, China) incubation at room temperature for 2 h the next day. Nuclei were stained by DAPI (4′,6-diamidino-2-phenylindole) (Beyotime, Shanghai, China). Then, cardiac fibroblasts were visualized and photographed with a fluorescence microscope (Nikon, Tokyo, Japan).

### 2.6. Measurement of Superoxide Formation

After treatment as the above description, the cardiac fibroblasts were incubated with dihydroethidium (DHE, 2 *μ*M, in the Krebs-HEPES buffer) at 37°C without light for 30 min. The level of superoxide in cells, presented as DHE fluorescence intensity, was detected with a laser confocal microscope (Leica, Wetzlar, Germany).

### 2.7. Assessment of Mitochondrial Respiration Function

The mitochondrial respiration oxygen consumption rate (OCR) was measured as the previous description by a Seahorse Extracellular Flux Analyzer (XF-96, Seahorse Bioscience, Santa Clara, CA, USA) [16]. After being plated in XF-96-well plates, the cardiac fibroblasts were subjected to siRNA transfection and treatment as described above. Then, the medium was changed into unbuffered DMEM containing glucose (10 mM), pyruvate (10 mM), and GlutaMAX (2 mM, Invitrogen, Carlsbad, CA, USA). Basal respiration was first obtained. After ATP synthesis was inhibited by oligomycin (Oligo, 2 *μ*g/mL), ATP generation during respiration was measured. Then, respiratory reserve capacity and maximal respiration were detected after uncoupler carbonyl cyanide 4-(trifluoromethoxy)phenylhydrazone (FCCP, 2 *μ*M) was added into the cells. Finally, rotenone and antimycin A (rot & AA, 4 *μ*M) were applied to completely block oxygen consumption.

### 2.8. Assessment of Mitochondrial Membrane Potential (Δ*ψ*m)

After treatment, the cardiac fibroblasts were incubated in JC-1 staining solution (Beyotime, Shanghai, China) at 37°C without light for 20 min. The level of mitochondrial membrane potential (Δ*ψ*m) was presented as the ratio of red fluorescence by aggregates of JC-1 to green fluorescence by monomers of JC-1, which were detected with a laser confocal microscope.

### 2.9. Animal Treatment

Male 8-week-old 129S1/SvImJ WT mice and SIRT3 KO mice were fed in the Experimental Animal Center of Nantong University according to the US National Institutes of Health Guidelines for Care and Use of Laboratory Animals (approval no. NTUERLAUA-20160224).

WT mice and SIRT3 KO mice were intraperitoneally administrated by NaHS (50 *μ*mol·kg^−1^·day^−1^) once daily, and normal saline (NS) was given as a control. After 2 weeks, mice were intraperitoneally injected with a mixture of ketamine (100 mg·kg^−1^) and xylazine (5 mg·kg^−1^) to induce anesthesia which was confirmed by loss of reflex to toe pinching. Then, thoracotomy was performed to expose the aortic arch, and transverse aortic constriction (TAC) was carried out by tying a 6-0 nylon suture ligature against a 26-gauge needle. The needle was withdrawn immediately to form an incomplete constriction. The mice with the same operating procedures without constriction served as sham controls. After surgery, all mice went on being treated by NaHS or NS for another 2 weeks [28]. During the experiments, systolic blood pressure (SBP) was monitored by a noninvasive blood pressure analysis system (Visitech Systems, Apex, NC, USA) with the tail-cuff method every week.

### 2.10. Determination of H_2_S Concentration in the Plasma and H_2_S Production in the Myocardium

The H_2_S level was determined using a H_2_S-specific microelectrode connected to a free radical analyzer (World Precision Instruments, Sarasota, FL, USA) in light of the previous description [16]. A standard curve was obtained by the current value in different concentrations (0, 0.5, 1, 2, 4, and 8 *μ*M) of Na_2_S stock solution. H_2_S in the myocardium was determined in tissue homogenates. The homogenates were incubated in the assay mixture (500 *μ*L) containing 460 *μ*L tissue homogenates, 20 *μ*L L-cysteine (10 mmol/L), and 20 *μ*L pyridoxal 5′-phosphate (2 mmol/L) at 37°C in tightly sealed vials for 30 min. Then, the plasma or homogenate was dropped into the reaction solution, and there was a significant change in the current value. The H_2_S level in the plasma or the myocardium can be calculated according to the standard curve.

### 2.11. Sirius Red Staining

After washing 3 times, myocardial paraffin sections about 5 *μ*m thick were stained with saturated picric acid-Sirius red without light for 30 min. Then, the nuclei were lightly stained with Mayer's hematoxylin staining solution. Finally, the collagen deposition in the myocardium was observed and photographed with a microscope. The ratio of the perivascular collagen area (PVCA) to the luminal area (LA) was calculated to assess the degree of perivascular fibrosis in the myocardium. The collagen volume fraction (CVF), as the ratio of the interstitial collagen area to the myocardial area, was statistically analyzed to evaluate the degree of interstitial fibrosis.

### 2.12. Quantitative Real-Time PCR

Expressions of mRNA were carried out by real-time PCR following the MIQE guidelines. Extracted RNA from cardiac fibroblasts or the myocardium with the TRIzol reagent (Takara, Kyoto, Japan) was subjected to reverse transcription according to the introduction of the PrimeScript™ RT Master Mix Kit (Takara, Kyoto, Japan). Amplification of cDNA was carried out three times in the PCR System (ABI 7500, ABI, Carlsbad, CA, USA) by SYBR (Takara, Kyoto, Japan) with 18S as the housekeeping gene. All sequences of the sense primers and antisense primers are listed below (Table 1).

### 2.13. Western Blots

Protein samples extracted from cardiac fibroblasts or the left ventricular myocardium were separated by 10% or 6% sodium dodecyl sulfate- (SDS-) polyacrylamide gel electrophoresis (PAGE). Then, the proteins in the gel were transferred onto membranes of polyvinylidene fluoride (PVDF) (Millipore, Billerica, MA, USA). After the membranes were blocked with 5% nonfat milk at room temperature for 2 h, the membranes with proteins were incubated overnight with an appropriate primary antibody for anti-SIRT3 (1 : 1000; Santa Cruz Biotechnology, St. Louis, MO, USA), anti-*α*-SMA, collagen I, collagen III (1 : 1000; Bioss, Beijing, China), anti-DRP1 (1 : 1000; Cell Signaling Technology, USA), anti-*β*-tubulin (1 : 3000; CMCTAG, Milwaukee, WI, USA), and anti-GAPDH (1 : 7000; Sigma-Aldrich, St. Louis, MO, USA) at 4°C. Next, membranes were incubated with horseradish peroxidase- (HRP-) labeled IgG at room temperature for 2 h. Enhanced chemiluminescence (ECL, Thermo Fisher Scientific Inc., Rockford, IL, USA) was dropped to visualize the protein blots.

### 2.14. Statistical Analysis

All data are shown as mean ± SEM and were analyzed by one-way ANOVA followed by the Bonferroni post hoc test (STATA 15.0). The values of *P* less than 0.05 were regarded as statistically significant.

## 3. Results

### 3.1. NaHS Inhibits Cell Proliferation but Enhances SIRT3 Transcription in Ang II-Stimulated Cardiac Fibroblasts

**Figure 1:**
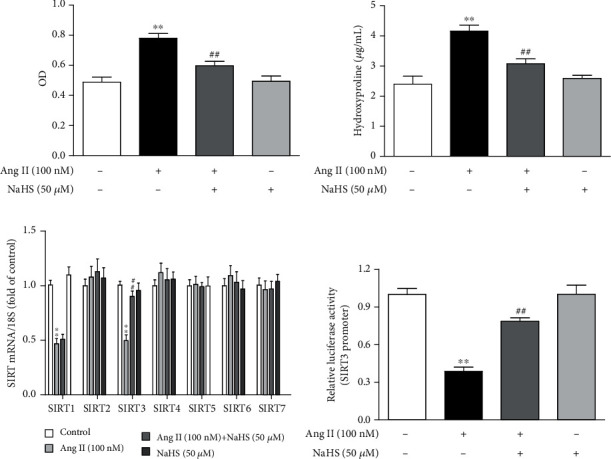
NaHS inhibits cell proliferation but enhances SIRT3 transcription in Ang II-stimulated cardiac fibroblasts. After pretreatment with NaHS (50 *μ*M) for 4 h, neonatal rat cardiac fibroblasts were stimulated by Ang II (100 nM) for 24 h. (a) The number of cardiac fibroblasts was detected with CCK-8. (b) The content of hydroxyproline in the cell culture medium was measured. (c) Expression of SIR2 family (SIRT1-7) mRNA was measured by real-time PCR. (d) After the SIRT3 promoter luciferase plasmid was transfected into cardiac fibroblasts for 24 h, cells were pretreated with NaHS (50 *μ*M) for 4 h followed by Ang II (100 nM) stimulation for another 24 h. Relative promoter activity of SIRT3 was detected with a dual-luciferase reporter assay system. ^∗∗^*P* < 0.01 as compared with untreated cells; ^##^*P* < 0.01 as compared with Ang II alone-stimulated cells. *n* = 6.

After NaHS (50 *μ*M) administration for 4 h followed by Ang II (100 nM) stimulation for another 24 h, cell proliferation was evaluated by cell count analysis and hydroxyproline secretion. NaHS pretreatment significantly reduced cell numbers (Figure 1(a)) and reduced hydroxyproline content in the cell culture medium (Figure 1(b)) after Ang II stimulation. These data indicated that NaHS inhibited Ang II-induced cardiac fibroblast proliferation.

Next, we assessed the possible role of SIR2 family members in the attenuated effect of NaHS on cardiac fibroblast proliferation. The mRNA expressions of all seven members, SIRT1-SIRT7, were detected by real-time PCR. SIRT1 and SIRT3 were significantly decreased after Ang II stimulation, while SIRT2, SIRT4, SIRT5, SIRT6, and SIRT7 remained unchanged. Moreover, the decreased SIRT3 was corrected by NaHS preadministration for 4 h. However, there was no significant change in SIRT1 by NaHS, suggesting that SIRT1 was possibly not critical in the inhibitory effect of NaHS on Ang II-induced cardiac fibroblast proliferation. It is worth noting that there was no alteration on SIR2 mRNA of NaHS-treated cardiac fibroblasts without Ang II stimulation (Figure 1(c)). Moreover, NaHS also increased SIRT3 promoter activity in Ang II-stimulated cardiac fibroblasts (Figure 1(d)). Taken together, NaHS enhanced SIRT3 transcription in the inhibitory effect on Ang II-induced cardiac fibroblast proliferation.

### 3.2. NaHS Inhibits Ang II-Stimulated Cardiac Fibroblast Proliferation via SIRT3

**Figure 2:**
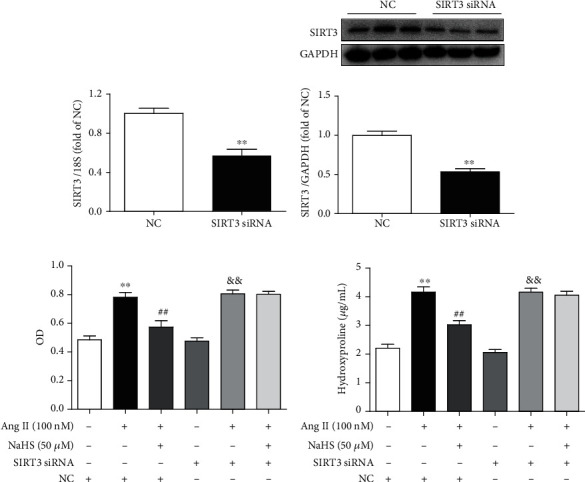
NaHS inhibits Ang II-stimulated cardiac fibroblast proliferation via SIRT3. (a, b) After SIRT3 siRNA or NC siRNA was transfected into neonatal rat myocardial fibroblasts for 48 h, expression of SIRT3 mRNA and protein was measured by real-time PCR and western blot, respectively. ^∗∗^*P* < 0.01 as compared with cells with NC siRNA transfection. *n* = 6. (c) After SIRT3 siRNA or NC siRNA was transfected into cardiac fibroblasts for 24 h, the cells were pretreated with NaHS (50 *μ*M) for 4 h followed by Ang II (100 nM) stimulation for another 24 h. The number of cardiac fibroblasts was detected with CCK-8. (d) The content of hydroxyproline in the cell culture medium was measured. ^∗∗^*P* < 0.01 as compared with untreated cells with NC siRNA transfection; ^##^*P* < 0.01 as compared with Ang II alone-stimulated cells with NC siRNA transfection; ^&&^*P* < 0.01 as compared with untreated cells with SIRT3 siRNA transfection. *n* = 6.

However, whether enhanced SIRT3 was crucial in the above effect remained to be elucidated. In our further study, SIRT3 was knocked down in cardiac fibroblasts by RNA interference technology. After specific SIRT3 siRNA transfection, SIRT3 was significantly reduced at both levels of mRNA and protein expressions (Figures 2(a) and 2(b)).

Then, cell numbers and hydroxyproline content of cardiac fibroblasts were assessed again after SIRT3 was silenced. Our study showed that NaHS pretreatment significantly decreased cell numbers and reduced hydroxyproline content in Ang II-stimulated cardiac fibroblasts. However, the inhibitory effect was unavailable after SIRT3 was knocked down (Figures 2(c) and 2(d)). These results suggested that NaHS inhibited Ang II-stimulated cardiac fibroblast proliferation in a SIRT3-dependent manner.

### 3.3. NaHS Suppresses Collagen Expression in Ang II-Stimulated Cardiac Fibroblasts via SIRT3

**Figure 3:**
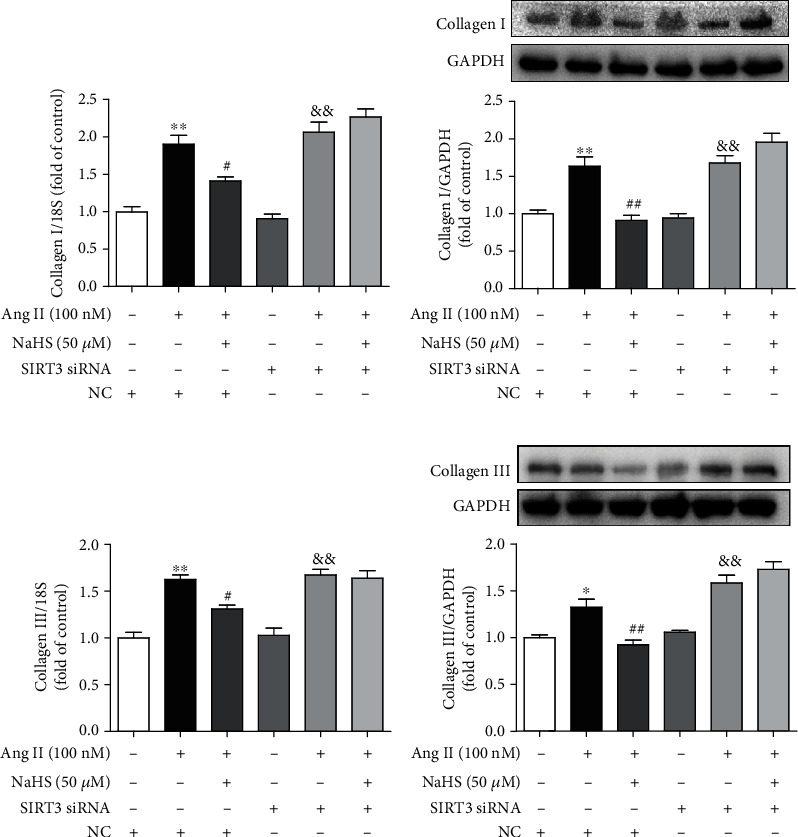
NaHS suppresses collagen expression in Ang II-stimulated cardiac fibroblasts via SIRT3. After SIRT3 siRNA or NC siRNA was transfected into neonatal rat cardiac fibroblasts for 24 h, the cells were pretreated with NaHS (50 *μ*M) for 4 h followed by Ang II (100 nM) stimulation for another 24 h. (a, b) Expression of collagen I mRNA and protein was measured by real-time PCR and western blot, respectively. (c, d) Expression of collagen III mRNA and protein was measured by real-time PCR and western blot, respectively. ^∗^*P* < 0.05 and ^∗∗^*P* < 0.01 as compared with untreated cells with NC siRNA transfection; ^#^*P* < 0.05 and ^##^*P* < 0.01 as compared with Ang II alone-stimulated cells with NC siRNA transfection; ^&&^*P* < 0.01 as compared with untreated cells with SIRT3 siRNA transfection. *n* = 6.

Next, the expression of collagen I and collagen III was also examined. We found that NaHS inhibited collagen I and collagen III expressions at both the mRNA and protein levels in Ang II-stimulated cardiac fibroblasts. However, the above protective effects by NaHS were absent after SIRT3 was silenced (Figure 3). It suggested that NaHS suppressed Ang II-stimulated cardiac fibroblast collagen expression in a SIRT3-dependent manner.

### 3.4. NaHS Inhibits *α*-SMA Expression in Ang II-Stimulated Cardiac Fibroblasts via SIRT3

**Figure 4:**
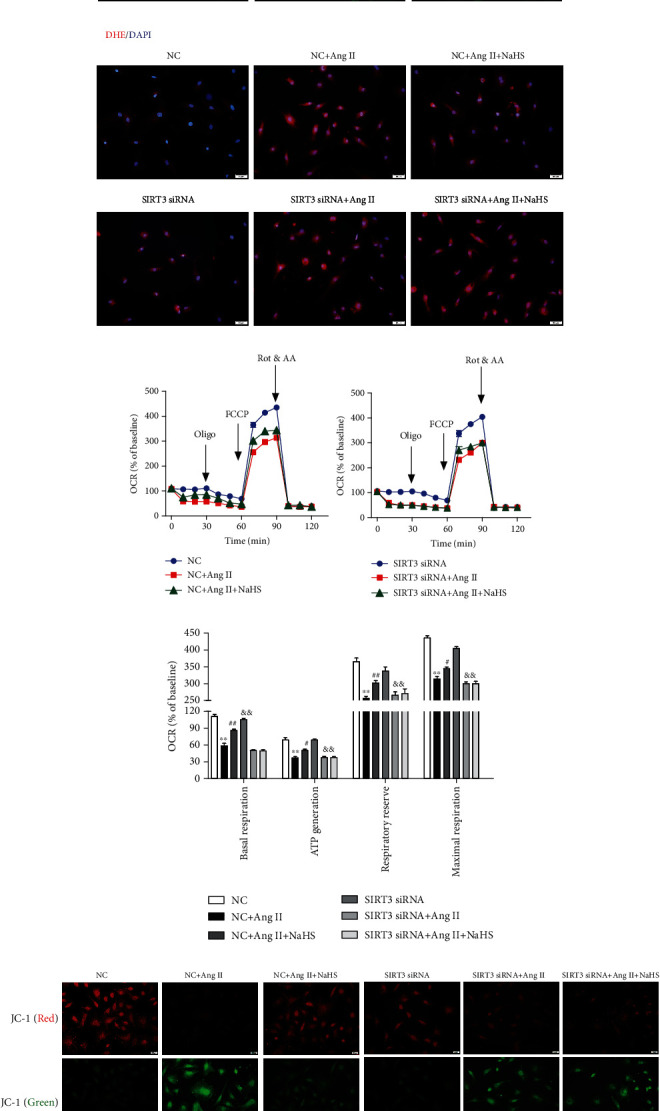
NaHS inhibits *α*-SMA expression and oxidative stress and improves mitochondrial respiration function and membrane potential in Ang II-stimulated cardiac fibroblasts via SIRT3. After SIRT3 siRNA or NC siRNA was transfected into neonatal rat cardiac fibroblasts for 24 h, the cells were pretreated with NaHS (50 *μ*M) for 4 h followed by Ang II (100 nM) stimulation for another 24 h. (a) Expression of *α*-SMA in cardiac fibroblasts was measured by immunofluorescence with Alexa Fluor 488 (green)-conjugated IgG. The nuclei were stained using DAPI (blue). Bar = 50 *μ*m. (b) ROS was detected with DHE staining. Bar = 50 *μ*m. (c) The mitochondrial respiration function of cardiac fibroblasts was measured. (d) Quantitative analysis of basal respiration, ATP generation, respiratory reserve capacity, and maximal respiratory. ^∗∗^*P* < 0.01 as compared with untreated cells with NC siRNA transfection; ^#^*P* < 0.05 and ^##^*P* < 0.01 as compared with Ang II alone-stimulated cells with NC siRNA transfection; ^&&^*P* < 0.01 as compared with untreated cells with SIRT3 siRNA transfection. *n* = 6. (e) Mitochondrial permeability potential was determined by JC-1 staining. Bar = 200 *μ*m.

The expression of *α*-SMA is commonly regarded as a robust and sensitive marker of cardiac fibroblast differentiation [4]. The present studies found that the *α*-SMA expression was enhanced after Ang II stimulation, which was attenuated by NaHS pretreatment. However, the above inhibitory effect of NaHS on *α*-SMA expression was obviously blunted if SIRT3 was knocked down (Figure 4(a)). It suggested that NaHS inhibited *α*-SMA expression in Ang II-stimulated cardiac fibroblasts via SIRT3.

### 3.5. NaHS Attenuates Oxidative Stress in Ang II-Stimulated Cardiac Fibroblasts via SIRT3

Previous studies have verified that excessive oxidative stress was a critical pathophysiological mechanism in several cardiovascular diseases including myocardial remodeling, atherosclerosis, myocardial ischemia-reperfusion injury, and diabetic cardiomyopathy [29, 30]. SIRT3, as a vital acetyl-lysine deacetylase, regulates mitochondrial function and reactive oxygen species (ROS) production [31]. Our present study found that NaHS alleviated DHE fluorescence intensity in Ang II-stimulated cardiac fibroblasts, suggesting that NaHS attenuated Ang II-stimulated ROS production. However, NaHS was no longer able to suppress DHE fluorescence intensity after SIRT3 was knocked down (Figure 4(b)). These data showed that NaHS attenuated oxidative stress in Ang II-stimulated cardiac fibroblasts via a SIRT3-dependent manner.

### 3.6. NaHS Improves Mitochondrial Respiration Function and Membrane Potential in Ang II-Stimulated Cardiac Fibroblasts via SIRT3

The above attenuated effects of NaHS on cardiac fibroblast proliferation suggested that mitochondrial function might also be improved by NaHS via SIRT3. To verify the hypothesis, the oxygen consumption rate (OCR) in Ang II-stimulated cardiac fibroblasts after NaHS administration with SIRT3 silencing was measured. Our study showed that NaHS significantly improved basal respiration, ATP generation, respiratory reserve capacity, and maximal respiration in Ang II-stimulated cardiac fibroblasts, which was unavailable after SIRT3 silencing (Figures 4(c) and 4(d)). These data suggested that NaHS improved mitochondrial respiration function in Ang II-stimulated cardiac fibroblasts via SIRT3.

Next, the mitochondrial membrane potential of cardiac fibroblasts was measured by JC-1 staining. Cells with strong red fluorescence intensity indicated the normal mitochondrial membrane potential, while green fluorescence indicated a decreased one. Our results showed that NaHS increased red, but decreased green, fluorescence intensity in Ang II-stimulated cardiac fibroblasts, whereas the effect was significantly alleviated after SIRT3 was knocked down (Figure 4(e)). It suggested that NaHS improved mitochondrial membrane potential in Ang II-stimulated cardiac fibroblasts via a SIRT3-dependent manner.

### 3.7. NaHS Decreases Blood Pressure but Restores H_2_S Levels and SIRT3 Expression in Mice with TAC

**Figure 5:**
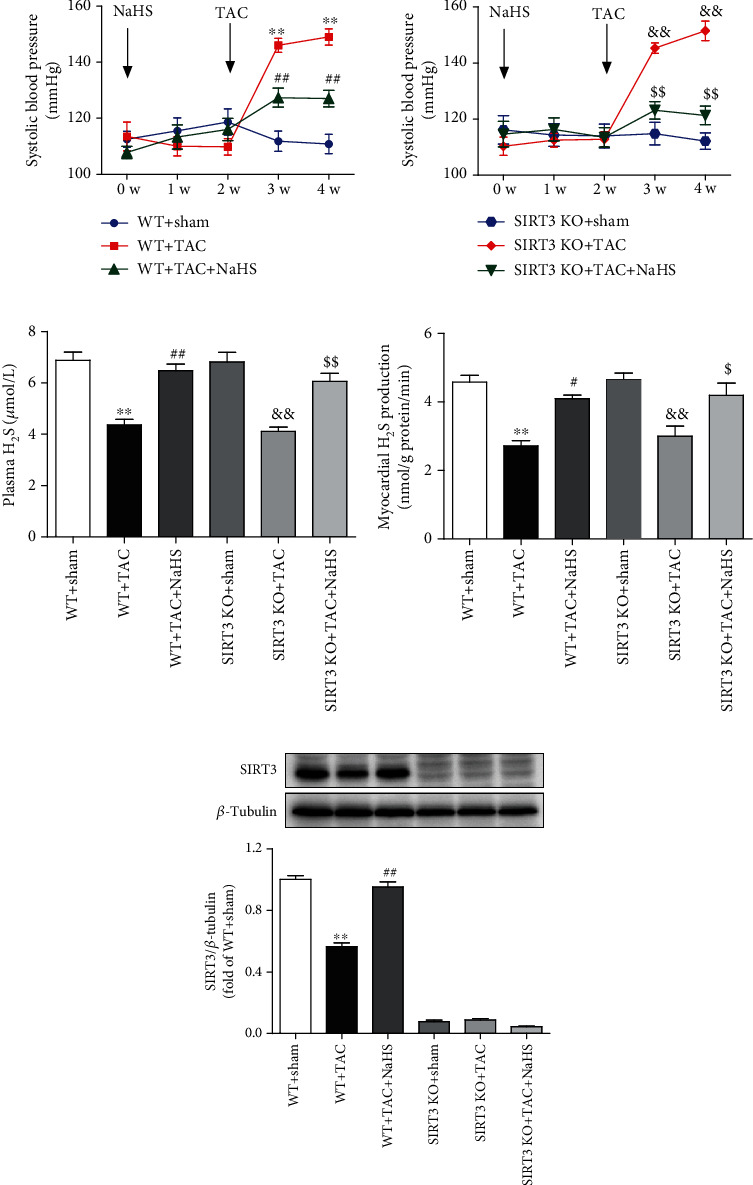
NaHS decreases blood pressure but restores H_2_S levels and SIRT3 expression in mice with TAC. After intraperitoneal injection by NaHS (50 *μ*mol·kg^−1^·day^−1^) or normal saline (NS) for 2 weeks, male wild-type (WT) mice and SIRT3 knockout (SIRT3 KO) mice were subjected to transverse aortic constriction (TAC) surgery. NaHS or NS was administrated for another 2 weeks. (a) The level of SBP in WT mice and SIRT3 KO mice was monitored every week after NaHS administration. (b) H_2_S concentration in the plasma was measured. (c) H_2_S production in the myocardium was detected. (d) Expression of SIRT3 protein in the myocardium was measured by western blot. ^∗∗^*P* < 0.01 as compared with WT+Sham; ^#^*P* < 0.05 and ^##^*P* < 0.01 as compared with WT+TAC; ^&&^*P* < 0.01 as compared with SIRT3 KO+Sham; ^$$^*P* < 0.01 as compared with SIRT3 KO+TAC. *n* = 6.

The above results showed that NaHS inhibited Ang II-induced cardiac fibroblast proliferation in a SIRT3-depended manner *in vitro*. Next, the role of SIRT3 in the protective effects of NaHS was investigated in mice. After TAC, SBP was significantly increased in WT and SIRT3 KO mice to the same extent in the next weeks. NaHS administration reduced SBP in both the WT and SIRT3 knockout mice (Figure 5(a)). H_2_S concentration in the plasma and H_2_S production in the myocardium were impaired after TAC, which was restored by the exogenous NaHS supplement in both WT mice and SIRT3 KO mice (Figures 5(b) and 5(c)). SIRT3 expression was decreased in the myocardium of WT mice after TAC, which was elevated by NaHS. And there was no SIRT3 expression in the myocardium of SIRT3 KO mice (Figure 5(d)). These data suggested that NaHS regulated blood pressure and H_2_S levels regardless of the presence or absence of the SIRT3 gene.

### 3.8. NaHS Ameliorates Perivascular and Interstitial Collagen Deposition in the Myocardium of WT Mice but Not SIRT3 KO Mice with TAC

**Figure 6:**
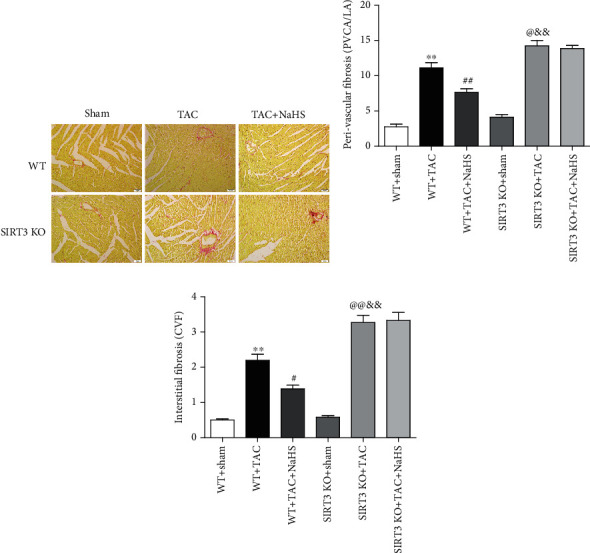
NaHS ameliorates collagen deposition in the myocardium of WT mice but not SIRT3 KO mice with TAC. After intraperitoneal injection by NaHS (50 *μ*mol·kg^−1^·day^−1^) or NS for 2 weeks, male WT mice and SIRT3 KO mice were subjected to TAC surgery. NaHS or NS was administrated for another 2 weeks. (a) Collagen deposition in the myocardium was stained with saturated picric acid-Sirius red. Bar = 50 *μ*m. (b) Perivascular fibrosis of the myocardium was assessed by the ratio of the perivascular collagen area (PVCA) to the luminal area (LA). (c) Interstitial fibrosis of the myocardium was assessed by the collagen volume fraction (CVF). ^∗∗^*P* < 0.01 as compared with WT+Sham; ^#^*P* < 0.05 or ^@^*P* < 0.05 and ^##^*P* < 0.01 or ^@@^*P* < 0.01 as compared with WT+TAC; ^&&^*P* < 0.01 as compared with SIRT3 KO+Sham. *n* = 6.

Perivascular fibrosis and interstitial fibrosis are the main manifestations of myocardial fibrosis [32]. Collagen, shown in red by Sirius red staining, was significantly suppressed by NaHS in WT mice but not SIRT3 KO mice with TAC (Figure 6(a)). Statistical analysis showed that after TAC, both the ratio of PVCA to LA and the CVF in SIRT3 KO mice were higher than that in WT mice. NaHS decreased the ratio of PVCA to LA and reduced CVF in WT mice with TAC, which was unavailable in SIRT3 KO mice (Figures 6(b) and 6(c)). It indicated that NaHS ameliorated perivascular and interstitial collagen deposition in the myocardium of WT mice but not SIRT3 KO mice with TAC.

### 3.9. NaHS Reduces Collagen and *α*-SMA Expressions in the Myocardium of WT Mice but Not SIRT3 KO Mice with TAC

**Figure 7:**
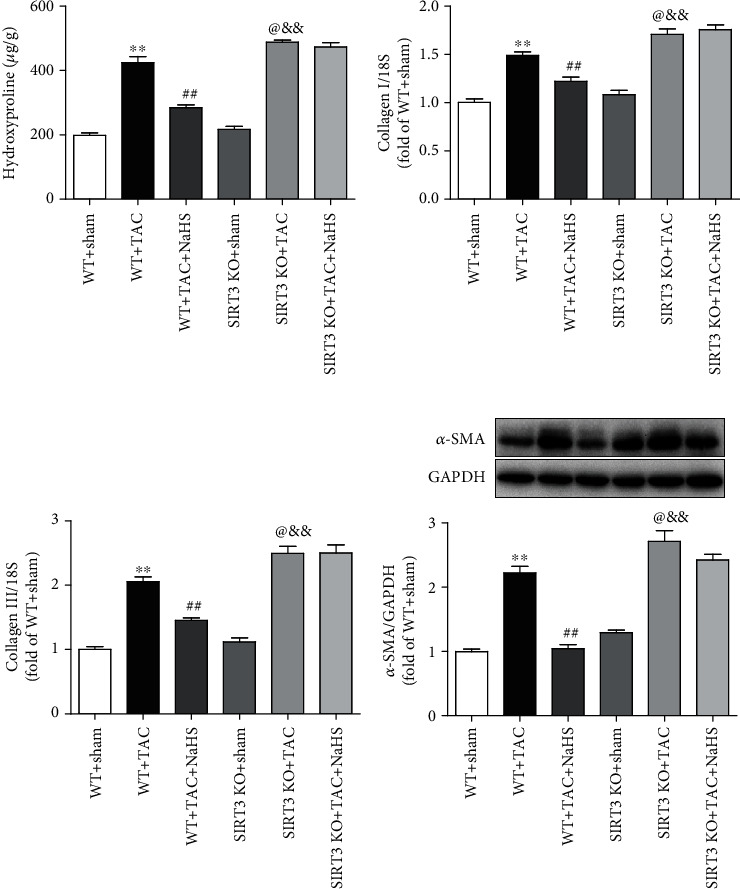
NaHS reduces collagen and *α*-SMA expressions in the myocardium of WT mice but not SIRT3 KO mice with TAC. After intraperitoneal injection by NaHS (50 *μ*mol·kg^−1^·day^−1^) or NS for 2 weeks, male WT mice and SIRT3 KO mice were subjected to TAC surgery. NaHS or NS was administrated for another 2 weeks. (a) The content of hydroxyproline in the myocardium was measured. (b, c) Expression of collagen I and collagen III mRNA in the myocardium was measured by real-time PCR. (d) Expression of *α*-SMA protein in the myocardium was measured by western blot. ^∗∗^*P* < 0.01 as compared with WT+Sham; ^##^*P* < 0.01 or ^@^*P* < 0.05 as compared with WT+TAC; ^&&^*P* < 0.01 as compared with SIRT3 KO+Sham. *n* = 8.

The data *in vitro* suggested that NaHS reduced collagen expression in Ang II-stimulated cardiac fibroblasts via a SIRT3-dependent manner. Next, the possible protective effect *in vivo* was further investigated. We found that after TAC, there was more hydroxyproline content and collagen expression in SIRT3 KO mice than in WT mice. NaHS reduced hydroxyproline content and collagen expression in the myocardium of WT mice with TAC. However, there was no inhibitory effect of NaHS on the above indexes in SIRT3 KO mice with TAC (Figures 7(a)–7(c)). Similarly, there was more *α*-SMA expression in SIRT3 KO mice with TAC than in WT mice. NaHS suppressed *α*-SMA expression in the myocardium of WT mice but not SIRT3 KO mice with TAC (Figure 7(d)). Taken together, NaHS alleviated myocardial fibrosis in mice with TAC via a SIRT3-dependent manner.

### 3.10. NaHS Restores DRP1 Expression in the Cardiac Fibroblasts with Ang II Stimulation and in the Myocardium of Mice with TAC via SIRT3

**Figure 8:**
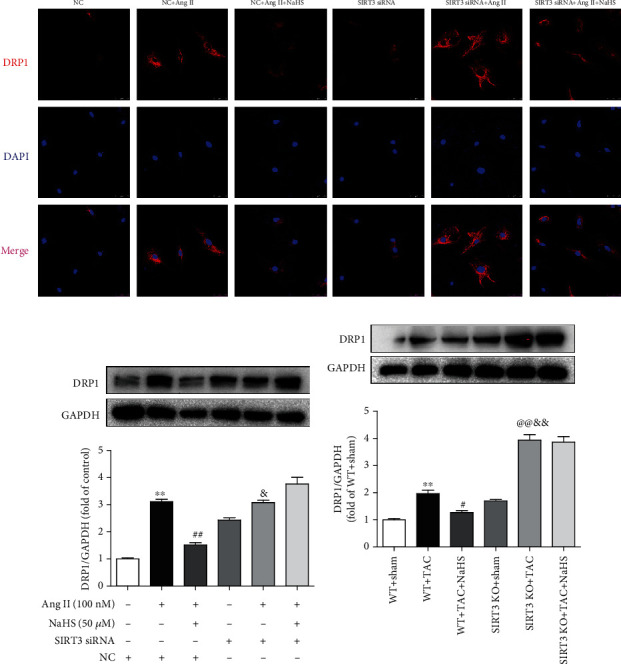
NaHS restores DRP1 expression in the cardiac fibroblasts with Ang II stimulation and in the myocardium of mice with TAC via SIRT3. (a) After SIRT3 siRNA or NC siRNA was transfected into neonatal rat cardiac fibroblasts for 24 h, the cells were pretreated with NaHS (50 *μ*M) for 4 h followed by Ang II (100 nM) stimulation for another 24 h. DRP1 expression in cardiac fibroblasts was detected by immunofluorescence with Cy3 (red)-conjugated IgG. The nuclei were stained using DAPI (blue). Bar = 25 *μ*m. (b) Expression of DRP1 protein was measured by western blot. ^∗∗^*P* < 0.01 as compared with untreated cells with NC siRNA transfection; ^##^*P* < 0.01 as compared with Ang II alone-stimulated cells with NC siRNA transfection; ^&^*P* < 0.05 as compared with untreated cells with SIRT3 siRNA transfection. *n* = 6. (c) After intraperitoneal injection by NaHS (50 *μ*mol·kg^−1^·day^−1^) or NS for 2 weeks, male WT mice and SIRT3 KO mice were subjected to TAC surgery. NaHS or NS was administrated for another 2 weeks. Expression of DRP1 protein in the myocardium was measured by western blot. ^∗∗^*P* < 0.01 as compared with WT+Sham; ^#^*P* < 0.05 or ^@@^*P* < 0.01 as compared with WT+TAC; ^&&^*P* < 0.01 as compared with SIRT3 KO+Sham. *n* = 6.

DRP1 is a protein associated with mitochondrial fission, which may induce structural damage and functional dysfunction of mitochondria [33]. Our present results showed that NaHS inhibited DRP1 expression in Ang II-stimulated cardiac fibroblasts. However, the inhibitory effect by NaHS was significantly weakened after SIRT3 was knocked down (Figures 8(a) and 8(b)). After TAC, there was more expression of DRP1 in the myocardium of SIRT3 KO mice than in WT mice. NaHS suppressed DRP1 expression in the myocardium of WT mice but not SIRT3 KO mice with TAC (Figure 8(c)). All these data verified that NaHS restored DRP1 expression in the cardiac fibroblasts with Ang II stimulation and in the myocardium of mice with TAC via a SIRT3-dependent manner.

## 4. Discussion

Myocardial fibrosis is the process of extracellular matrix remodeling to significantly increase myocardial stiffness and eventually results in heart failure and even sudden cardiac death [34]. The H_2_S supplement might have protective effects on myocardial ischemia-reperfusion injury, cardiac infarction, arrhythmia, cardiac hypertrophy, heart failure, and diabetic cardiomyopathy [35–37]. It is noted that H_2_S suppressed cardiac fibroblast proliferation by inhibiting K^+^ currents or channels and blocking the transformation of atrial fibroblasts into myoblasts [38]. NaHS administration ameliorated myocardial fibrosis via inhibiting cell aging in diabetic rats [39]. H_2_S alleviated myocardial fibrosis and restored cardiac function in both the CSE KO and WT mice with myocardial infarction [40]. In our present experiment, we verified that NaHS inhibited cell numbers, hydroxyproline content, *α*-SMA expression, and collagen production but enhanced SIRT3 expression in the cardiac fibroblasts and the myocardium. It suggested the inhibitory effect of H_2_S on both Ang II-induced cardiac fibroblast proliferation and TAC-induced myocardial fibrosis. Moreover, NaHS equally reduced blood pressure but enhanced the H_2_S level in all mice, suggesting that the failure of NaHS to inhibit myocardial fibrosis in SIRT3 KO mice is due to SIRT3 deficiency but not blood pressure.

The key role of SIRT3 in mitochondrial function has been intensively investigated. However, the possible role of SIRT3 on the protective effects of H_2_S against myocardial fibrosis was unknown. Our previous research studies have shown that H_2_S increased SIRT3 expression in human umbilical vein endothelial cells and cardiomyocytes [16, 18, 41]. Other groups found that H_2_S improved cardiac energy substrate metabolism via SIRT3 in db/db mice [42, 43]. H_2_S activated SIRT3 through S-sulfhydration to attenuate cisplatin-induced acute kidney injury [44]. H_2_S also attenuated hydrogen peroxide-induced NLR family pyrin domain-containing protein 3 (NLRP3) inflammasome activation via SIRT3 in macrophages [45]. Moreover, we verified that SIRT3 deficiency exacerbated diabetic cardiomyopathy and delayed diabetic skin wound healing [46, 47]. In our present experiment, mRNA expression of SIRT1 and SIRT3 was decreased, while other subtypes of the SIR2 family did not change significantly in cardiac fibroblasts with Ang II stimulation. Moreover, NaHS did increase SIRT3 transcription. Therefore, SIRT3 was focused on the possible pathway of the protective effect on myocardial fibrosis next. We found that there was more serious fibrosis in SIRT3 KO mice after TAC, suggesting the protective role of SIRT3 in TAC-induced myocardial fibrosis. Cardiac fibroblasts with SIRT3 knockdown and mice with SIRT3 deficiency further verified the protective effect on myocardial fibrosis by H_2_S via a SIRT3-depended manner. It is beneficial to clarify the mechanism of H_2_S against myocardial fibrosis.

It is worth noting that the researchers know little about the detailed mechanism of how H_2_S regulated SIRT3 transcription. Our previous study verified that H_2_S increased activator protein 1 (AP-1) binding activity with the SIRT3 promoter to enhance SIRT3 transcription in hydrogen peroxide- (H_2_O_2_-) stimulated EA.hy926 endothelial cells [18]. Others demonstrated that the protective effect of H_2_S in paraquat-induced liver injury was at least partly attributed to nuclear factor erythroid 2-related factor 2- (Nrf2-) dependent SIRT3 gene transcription [48]. H_2_S can induce S-sulfhydration on specific cysteine residues of target proteins to alter protein function and signal transduction [13]. Some studies found that H_2_S S-sulfhydrated c-Jun of AP-1 to enhance the transcriptional activity on SIRT3, which contributed to decrease ROS production in H_2_O_2_-stimulated macrophages [45]. However, the mechanism of how H_2_S regulates SIRT3 transcriptional activity during myocardial fibrosis is not known at present and needs to be confirmed in further studies.

SIRT3 plays a vital role in mitochondrial biosynthesis and oxidative stress, which might be critical in the process of myocardial fibrosis [49]. Several studies also indicated that H_2_S was capable of attenuating oxidative stress and mitochondrial dysfunction [16, 50]. H_2_S inhibited oxidative stress in the myocardium of chronic heart failure [51]. Studies have shown that impaired mitochondrial permeability transition pores reduced mitochondrial membrane potential depolarization and inhibited the activation of proapoptotic proteins [52]. It has been reported that H_2_S effectively improved mitochondrial membrane potential in high glucose-stimulated human umbilical vein endothelial cells [53]. This may be one of the important mechanisms for protection against mitochondrial function by H_2_S [54]. We found that NaHS significantly inhibited Ang II-induced ROS production, improved mitochondrial respiration function, and restored membrane potential in a SIRT3-dependent manner. However, the detailed mechanism of the SIRT3-mediated inhibitory effect on oxidative stress by H_2_S was not known well now.

Mitochondria are highly dynamic organelles undergoing cycles of fusion and fission to modulate the morphology, distribution, and function. DRP1, a key protein to regulate mitochondrial fission, is related to clearing the damaged mitochondria and maintaining the process of cellular and organ dynamics [55]. However, an excessive increase of DRP1 will break the balance between mitochondrial fusion and fission to cause mitochondrial dysfunction. Therefore, mitochondrial fission manipulated by targeting DRP1 has been an appealing therapeutic strategy for cytoprotection [56]. We found that NaHS corrected the excessive enhancement of DRP1 in both the cardiac fibroblasts with Ang II stimulation and the myocardium with TAC via a SIRT3-mediated pathway. It clarified a novel mechanism for H_2_S and proposed an alternative approach for mitochondrial function protection.

**Figure 9:**
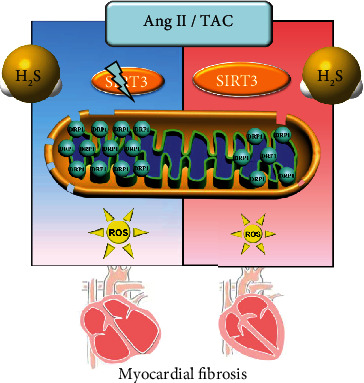
Illustration of the mechanism of protective effects on myocardial fibrosis by H_2_S. H_2_S enhanced SIRT3 transcription, decreased the DRP1 level, ameliorated mitochondrial membrane rupture, suppressed oxidative stress, and alleviated Ang II-induced cardiac fibroblast proliferation and TAC-induced myocardial fibrosis. However, these protective effects of H_2_S were unavailable if SIRT3 was silenced in cells or deficient in mice. It suggested that H_2_S attenuated myocardial fibrosis through oxidative stress inhibition via a SIRT3-dependent manner.

In conclusion, NaHS, a H_2_S donor, enhanced SIRT3 transcription and decreased the DRP1 level to possibly ameliorate mitochondrial membrane rupture, suppress oxidative stress, and alleviate Ang II-induced cardiac fibroblast proliferation and TAC-induced myocardial fibrosis. An illustration of the mechanism is outlined in Figure 9. However, these protective effects of H_2_S were unavailable if SIRT3 was silenced in cells or deficient in mice. These results have shed new light on the molecular mechanism responsible for the cardioprotective effect of H_2_S against myocardial fibrosis through SIRT3 activation, which might propose a novel strategy for myocardial fibrosis prevention and treatment.

## Figures and Tables

**Table 1:** Sequences of primers.

Gene	Sense primer	Antisense primer
Rat SIRT1	5′-CACCAGAAAGAACTTCACCACCAGA-3′	5′-ACCATCAAGCCGCCTACTAATCTG-3′
Rat SIRT2	5′-AGGGACAAGGAGCAGGGTTC-3′	5′-GAAGAGAGACAGCGGCAGGAC-3′
Rat SIRT3	5′-GAGGTTCTTGCTGCATGTGGTTG-3′	5′-AGTTTCCCGCTGCACAAGGTC-3′
Rat SIRT4	5′-TTGTGCCAGCAAGTCCTCCTC-3′	5′-GTCTCTTGGAAAGGGTGATGAAGC-3′
Rat SIRT5	5′-TCCAGCGTCCACACGAAACC-3′	5′-AACACCAGCTCCTGAGATGATGAC-3′
Rat SIRT6	5′-GCTGGAGCCCAAGGAGGAATC-3′	5′-AGTAACAAAGTGAGACCACGAGAG-3′
Rat SIRT7	5′-GAGCCAACCCTCACCCACATG-3′	5′-ACGCAGGAGGTACAGACTTCAATG-3′
Rat collagen I	5′-AGGGTCATCGTGGCTTCTCT-3′	5′-CAGGCTCTTGAGGGTAGTGT-3′
Rat collagen III	5′-AGCGGAGAATACTGGGTTGA-3′	5′-GATGTAATGTTCTGGGAGGC-3′
Mouse collagen I	5′-AAGAAGACATCCCTGAAGTCA-3′	5′-TTGTGGCAGATACAGATCAAG-3′
Mouse collagen III	5′-TTGGGATGCAGCCACCTTG-3′	5′-CGCAAAGGACAGATCCTGAG-3′
18S	5′-AGTCCCTGCCCTTTGTACACA-3′	5′-CGATCCGAGGGCCTCACTA-3′

## Data Availability

The data used to support the finding of this study are available from the corresponding author upon request.
